# Antimicrobial effects of catnip (*Nepeta catari*a L.) essential oil against canine skin infection pathogens

**DOI:** 10.14202/vetworld.2024.585-592

**Published:** 2024-03-08

**Authors:** Glenn Neville Borlace, Ranee Singh, Supawadee Seubsasana, Pranom Chantaranothai, Eakachai Thongkham, Jareerat Aiemsaard

**Affiliations:** 1 Department of Pharmaceutical Chemistry, Faculty of Pharmaceutical Sciences, Khon Kaen University, Khon Kaen 40002, Thailand; 2 Division of Pharmacology and Toxicology, Faculty of Veterinary Medicine, Khon Kaen University, Khon Kaen 40002, Thailand; 3 Division of Pharmaceutical Sciences, Faculty of Pharmacy, Thammasat University, Pathum Thani 12120, Thailand; 4 Department of Biology, Faculty of Science, Khon Kaen University, Khon Kaen 40002, Thailand

**Keywords:** antimicrobial activity, canine dermatitis, catnip, *Nepeta cataria*

## Abstract

**Background and Aim:**

Catnip essential oils have antimicrobial effects against bacteria, yeast, and fungi; however, there is limited information regarding their antimicrobial activity against pathogens that cause canine skin infections. This study aimed to identify the phytochemical constituents of catnip essential oil and assay its antimicrobial activity against *Staphylococcus pseudintermedius*, *Malassezia pachydermatis*, *Microsporum canis*, *Microsporum gypseum*, *Microsporum gallinae*, and *Trichophyton mentagrophytes*.

**Materials and Methods:**

Catnip essential oil was extracted by hydrodistillation, and its chemical constituents were analyzed by gas chromatography–mass spectrometry (GC–MS). *In vitro* antimicrobial activity was investigated using broth microdilution and time-kill tests. To evaluate the effect of catnip essential oil on microbial morphology and cell membrane integrity, scanning electron microscopy (SEM) and leakage studies were conducted.

**Results:**

GC–MS analysis revealed that the principal components of catnip essential oil were *cis*- and *trans*-nepetalactone (57.09% of peak area), *trans*-*, cis*-nepetalactone (39.69% of peak area), *trans*-caryophyllene (1.88% of peak area), and caryophyllene oxide (1.34% of peak area). The minimum inhibitory concentration, minimum bactericidal concentration, and minimum fungicidal concentration values determined by broth microdilution ranged from 0.0625 mg/mL to 4.0 mg/mL. Time-kill testing showed that the germicidal effects of catnip essential oil were time and concentration-dependent, respectively. Environmental SEM and cell leakage analysis indicated that catnip essential oil disrupted the integrity of cell membranes in the tested microorganisms.

**Conclusion:**

Catnip essential oil has potential as an alternative antimicrobial against a wide range of canine skin infection pathogens, including *S. pseudintermedius, M. pachydermatis, Mi. canis, Mi. gypseum, Mi. gallinae*, and *T. mentagrophytes*.

## Introduction

Infectious dermatitis in companion animals is a common disease caused by several bacteria, yeasts, and filamentous fungi. The primary pathogen of canine pyoderma is *Staphylococcus pseudintermediu*s, which can infect the skin, ear canal, and urinary tract of dogs as well as their respiratory and reproductive systems [1]. Methicillin-resistant *S. pseudintermedius* strains have been reported in many animals, including dogs, cats, horses, and donkeys [2]. *Malassezia pachydermatis* is a skin commensal and opportunistic pathogen that primarily causes *Malassezia* dermatitis and otitis externa in immunocompromised animals [3]. Dermatophyte molds are also a major cause of infectious dermatitis. The soil-associated dermatophyte *Microsporum gypseum* is an opportunistic infectious fungus that can be transmitted from the environment to an animal, leading to dermatophytosis [4]. Many zoophilic dermatophyte species, such as *Microsporum canis*, *Microsporum gallinae*, and *Trichophyton mentagrophytes*, can also be transmitted to humans [5, 6]. Managing these infections can be difficult due to increased rates of antifungal drug resistance among clinical isolates, particularly terbinafine and azoles [7].

Catnip (*Nepeta cataria* L.), a member of the mint family, contains feline attractants that have a soothing effect on cats and other felids. In addition, catnip extracts have antioxidant, analgesic, and anti-inflammatory properties with potential for therapeutic and medicinal purposes [8]. Catnip’s essential oil contains terpenoid nepetalactones, which are responsible for its soothing and insect-repelling effects [9]. Essential oils and extracts of catnip of catnip have been reported to have antibacterial [10–12] and antifungal activities [13, 14]. However, catnip essential oil has limited antimicrobial activity against animal pathogens that cause dermatitis.

This study aimed to identify the phytochemical constituents of catnip essential oil and assay its antimicrobial activity against *S. pseudintermedius*, *M. pachydermatis*, *Mi. canis*, *Mi. gypseum*, *Mi. gallinae*, and *T. mentagrophytes*.

## Materials and Methods

### Ethical approval

This study used *in vitro* experiments; therefore, ethical approval was not necessary for this study.

### Study period and location

This study was conducted from January to September 2023 at the Faculty of Veterinary Medicine, Khon Kaen University, Thailand.

### Plant sample preparation and extraction

Three-month-old catnip aerial parts were collected from local agricultural plots in Khon Kaen Province, Thailand. Botanical identification was confirmed, and plant specimens were preserved in the Khon Kaen University Herbarium (herbarium voucher number: J. Aiemsaard *et al*. 03). Fresh leaves were washed with distilled water, dried at 37°C, and ground to powder. A Clevenger apparatus was used in the hydrodistillation process. The process was carried out at 90–95°C for 24 h, and the obtained product was mixed with sodium sulfate (Sigma-Aldrich, Germany) and n-hexane (Brightchem Sdn Bhd, Malaysia). We collected the oil phase using a separatory funnel and filtered through Whatman filter paper No. 1. Solvent was evaporated at 55°C using a rotary evaporator (Heidolph, Germany) [9].

### Gas chromatography–mass spectrometry (GC–MS)

A 50 μL aliquot of catnip essential oil was diluted with 950 μL of methyl tert-butyl ether. GC–MS analysis was performed using an Agilent 6890N gas chromatograph and a 5973 Mass Selective Detector (Agilent Technologies, Inc., USA) equipped with a DB-5MS, 5% phenyl 95% dimethyl-poly siloxane fused-silica capillary column (30 m 0.25 mm, film thickness 0.25 μm performed using). Helium was used as the carrier gas at a constant flow rate of 1 mL/min; the injection volume (split mode) was 2 μL. The temperature was programmed to start at 70°C and then increased at a rate of 2°C/min to a maximum temperature of 220°C for 10 min. The inlet and ion source temperatures were 230°C and 280°C, respectively. We compared the mass spectra of the chemical constituents of catnip essential oil with mass spectral libraries (Wiley 7n.1, New Jersey, USA) [9].

### Microbial culture

*Staphylococcus pseudintermedius* American Type Culture Collection (ATCC) 49051, *M. pachydermatis* ATCC 14522, and *Mi. gallinae* ATCC 90749 were purchased from the ATCC (Corporate Office, University Boulevard Manassas, Virginia, USA). *Mi. canis* Department of Medical Sciences Thailand (DMST) 29297, *Mi. gypseum* DMST 21146, and *T. mentagrophytes* DMST 19735 were obtained from the DMST, Nonthaburi, Thailand. Bacteria and yeast were cultured in Mueller–Hinton broth (MHB) and Sabouraud dextrose broth (SDB; Becton Dickinson, France) at 37°C for 24 h. All dermatophytes were cultured on Sabouraud dextrose agar (SDA; Becton Dickinson) at 37°C for 7 days. Phosphate-buffered saline (PBS; pH 7.2) was then added to the plates and fungal hyphae were collected using a triangular glass rod spreader. Aerobic plate counts were used to verify the concentrations of the microbial suspensions utilized in the susceptibility tests [15–17].

### Broth microdilution test

The assay was performed according to the Clinical and Laboratory Standard Institute guidelines [15–17] with some modifications. Briefly, the catnip essential oil was dissolved in dimethyl sulfoxide (DMSO; Sigma-Aldrich) and serially two-fold diluted with MHB (bacteria) or SDB (fungi) in 96-well round-bottomed microtiter plates (Corning Incorporated, USA). *S. pseudintermedius* (1 × 10^6^ colony-forming units [CFU]/mL) or fungal (2 × 10^3^ CFU/mL) inocula were added to the wells separately. Bacterial and yeast microtiter plates were incubated at 37°C for 24 h and mold microtiter plates were incubated at 30°C for 72 h. Culture broths with and without microbial suspensions were used as growth control wells. The minimum inhibitory concentration (MIC) was the lowest essential oil concentration that inhibited visible growth after the incubation period. The minimum bactericidal concentration (MBC) and minimum fungicidal concentration (MFC) were the lowest concentrations of essential oil that inhibited the subsequent growth of bacteria on Mueller–Hinton agar (MHA; Becton Dickinson) and fungi on SDA. Standard antimicrobial controls were cephalexin and ketoconazole (Sigma-Aldrich), respectively.

### Time-kill assay

The time-kill assay was performed according to the method previously described by Punareewattana *et al*. [18] with some modifications. Briefly, 100 μL inocula containing 1 × 10^7^ CFU/mL of *S. pseudintermedius* and *M. pachydermatis* and 1 × 10^3^–1 × 10^4^ CFU/mL of filamentous fungi were mixed with 900 μL of diluted catnip essential oil (in PBS) to give final concentrations of after incubation at 30°C for 1, 2, 4, 6, and 8 h, the cultures were 10-fold diluted with PBS, and 100 μL of the 10^−1^–10^−3^ dilutions were spread onto MHA or SDA plates and incubated at 37°C for 24 h (for bacteria and yeast) or 30°C for 72 h (for filamentous fungi). The number of colonies recovered is expressed as log_10_ reduction in the number of viable cells (CFU/mL). The negative growth control and diluent control wells contained PBS and DMSO (62.5 µL/mL), respectively.

### Scanning electron microscopy (SEM)

Environmental SEM (E-SEM, Thermo Scientific™ Quattro-S E-SEM, Thermo Fisher Scientific Inc., USA) was used to examine the effect of catnip essential oil on microbial morphology. In the time-kill assay, the microbial cells were treated for 10 min with the minimum concentration of catnip essential oil that produced a 5-log_10_ reduction for the bacterium and yeast or a 3-log_10_ reduction for the molds within 1 h. Cells were then washed 3 times with sterile distilled water and centrifuged at 3000× *g* for 5 min [19]. The obtained pellets were transferred to carbon conductive tabs and allowed to air dry before being observed under E-SEM at 2,500× (for fungi) and 50,000× (for bacteria) in a high vacuum environment at 5–10 kV.

### Leakage study

Microbial suspensions were centrifuged at 3500× *g* for 10 min. The obtained pellets were washed 3 times with sterile distilled water and resuspended in catnip essential oil at the same concentration as that used in the E-SEM study. Pellets resuspended in 5% DMSO served as a leakage control. After 1, 2, 3, 4, 5, and 6 h, the samples were centrifuged at 3500× *g* for 10 min. Membrane integrity was determined using a microplate spectrophotometer (Epoch™ 2, BioTek Instruments, Inc., USA) by measuring the ultraviolet absorbance of the supernatant at 260 nm. Essential oil without microorganisms was used as the blank [20].

## Results

### Chemical constituents of catnip essential oil

**Figure-1:**
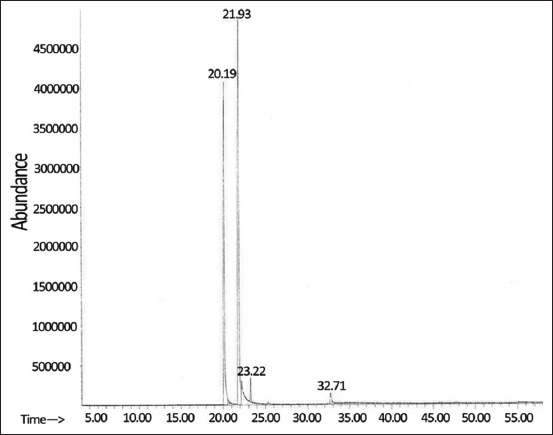
Gas chromatography–mass spectrometry spectrum of catnip essential oil. Retention times correspond to *trans, cis*-nepetalactone (20.19 min), *cis, trans*-nepetalactone (21.93 min), *trans*-caryophyllene (23.22 min), and caryophyllene oxide (32.71 min).

The yield of catnip essential oil obtained from hydrodistillation was 0.41% of the dry plant weight. The GC–MS analysis (Figure-1) revealed that two nepetalactone isomers were the major constituents in the extracted oil (totaling 96.78% of the peak area), along with *trans*-caryophyllene and caryophyllene oxide (1.88% and 1.34% of the peak area, respectively) as minor constituents (Table-1). At a retention time of 20.19 min (39.69% of the peak area), the nepetalactone isomers in catnip essential oil were *trans-, cis*-nepetalactone (*E, Z*-nepetalactone) and *cis-, trans*-nepetalactone (*Z, E*-nepetalactone) at a retention time of 21.93 min (57.09% of the peak area).

**Table 1:** Chemical constituent of catnip essential oil from gas chromatography–mass spectrometry analysis.

Chemical	Molecular formula	Retention time (min)	% of peak area
*trans, cis*-Nepetalactone	C_10_H_14_O_2_	20.19	39.69
*cis, trans*-Nepetalactone	C_10_H_14_O_2_	21.93	57.09
*trans*-Caryophyllene	C_15_H_24_	23.22	1.88
Caryophyllene oxide	C_15_H_24_O	32.71	1.34

### Antimicrobial activity

Table-2 shows the catnip essential oil broth microdilution assay results. The MIC/MBC value of cephalexin against *S. pseudintermediu*s was 0.125 g/mL, indicating that it was sensitive to cephalexin according to Clinical and Laboratory Standard Institute breakpoints [21]. The control antifungal ketoconazole was more effective against *M. pachydermatis* (MIC/MFC 0.0078 g/mL) than against filamentous fungi (MIC 0.50–8.00 μg/mL and MFC 0.50–16.00 μg/mL). MIC, MBC, and MFC values of catnip essential oil ranged from 0.0625 to 4.0 mg/mL. *M. pachydermatis* (MIC 0.0625 mg/mL), followed by *Mi. gypseum* (MIC 0.25 mg/mL) and *T. mentagrophytes* (MIC 0.25 mg/mL), was the most sensitive to catnip essential oil. *S. pseudintermedius*, *Mi. canis*, and *Mi. gallinae* had the highest MIC (1 mg/mL). The MBC and MFC values were the same or higher than their respective MICs. For *M. pachydermatis* and *T. mentagrophytes*, the MFC values were the same as their respective MICs; however, for *Mi. gallinae*, the MFC value was two-fold higher than their respective MICs; for *S. pseudintermedius* and *Mi. canis*, the MBC/MFC values were four-fold higher than their respective MICs; and for *Mi. gypseum*, the fungicidal concentration was eight-fold higher than their respective MICs. Three filamentous fungi (*Mi. canis*, *Mi. gypseum*, and *T. mentagrophytes*) showed substantially lower MIC and MFC values for catnip essential oil than those for ketoconazole.

**Table 2:** Antimicrobial activity of catnip essential oil and antimicrobial drugs against microorganisms causing dermatitis in animals.

Microorganism	Catnip essential oil (mg/mL)		Cephalexin (μg/mL)		Ketoconazole (μg/mL)	
		
	MIC	MBC/MFC	MIC	MBC	MIC	MBC
*Staphylococcus pseudintermedius* ATCC 49051	1.00	4.00	0.125	0.125	NA	NA
*Malassezia pachydermatis* ATCC 14522	0.0625	0.0625	NA	NA	0.0078	0.0078
*Microsporum canis* DMST 29297	1.00	4.00	NA	NA	4.00	16.00
*Microsporum gypseum* DMST 21146	0.25	2.00	NA	NA	8.00	16.00
*Microsporum gallinae* ATCC 90749	1.00	2.00	NA	NA	0.50	0.50
*Trichophyton mentagrophytes* DMST 19735	0.25	0.25	NA	NA	4.00	8.00

NA=Not applicable, Values represent MIC=Minimum inhibitory concentration, MBC=Minimum bactericidal concentration, MFC=Minimum fungicidal concentration collected from triplicate experiments. DMST=Department of Medical Sciences Thailand, ATCC=American Type Culture Collection

### Time-kill kinetics

**Figure-2:**
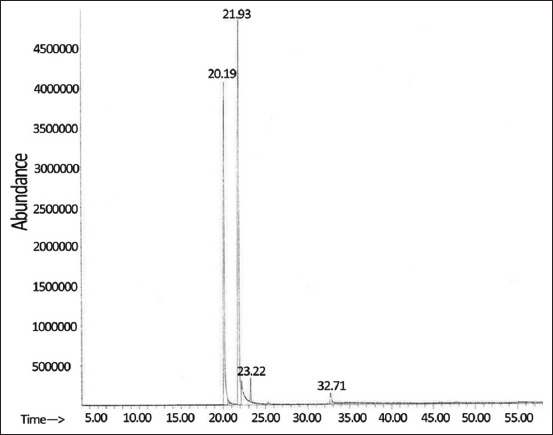
Time-kill kinetics of catnip essential oil against *Staphylococcus pseudintermedius* ATCC 49051 (1 × MIC = 1 mg/mL) (a), *Malassezia pachydermatis* ATCC 14522 (1 × MIC = 0.0625 mg/mL) (b), *Microsporum canis* DMST 29297 (1 × MIC = 1 mg/mL) (c), *Microsporum gypseum* DMST 21146 (1 × MIC = 0.25 mg/mL) (d), *Microsporum gallinae* ATCC 90749 (1 × MIC = 1 mg/mL) (e), and *Trichophyton mentagrophytes* DMST 19735 (1 × MIC = 0.25 mg/mL) (f). Control=normal saline solution. Values represent the mean of triplicate experiments with error bar (SD). DMST=Department of Medical Sciences Thailand, ATCC=American Type Culture Collection, SD=Standard deviation.

Figure-2 illustrates the results of the time-kill testing of catnip essential oil. Overall, the catnip essential oil produced dose- and time-dependent bactericidal and fungicidal effects against all examined microbial cells, with higher concentrations and longer contact times at lower concentrations showing increasing antimicrobial effects. The catnip essential oil reduced the number of viable *S. pseudintermedius* by 1.7-log_10_ after 8 h at 1 MIC (1 mg/mL). It eradicated the bacterium (a 7-log_10_ reduction) after 6 h at 4 × MIC and after 1 h at 16 × MIC (Figure-2a). The effects on *M. pachydermatis* were very similar to those of *S. pseudintermedius*. Catnip essential oil decreased the number of viable *M. pachydermatis* by 1.7-log_10_ after 8 h at 1 × MIC (0.0625 mg/mL) and eradicated the yeast after 6 h at 4 × MIC and after 1 h at 16 × MIC (Figure-2b).

The antifungal effects against each dermatophyte species were slightly different. Against *Mi. canis*, catnip essential oil reduced the number of viable cells by approximately 1-log_10_ after 8 h at 1 × MIC (1 mg/mL) and 4 × MIC and eradicated it (3.5-log_10_ reduction) after 1 h at 16 × MIC. *Mi. gypseum* was relatively unaffected by catnip essential oil at 1 × MIC (0.25 mg/mL) with a 0.3-log_10_ reduction in the number of viable cells after 8 h. Increasing the concentration to 4 MIC resulted in a 1.3-log_10_ reduction at 8 h, but the filamentous fungus was only eradicated after 2 h at 16 × MIC (4.5-log_10_ reduction). Catnip essential oil was able to reduce the number of viable *Mi. gallinae* by 1.3-log_10_ after 8 h at 1 × MIC (1 mg/mL) and eliminated the fungus (4-log_10_ reduction) after 6 h at 4 × MIC and 2 h at 16 × MIC. Finally, catnip essential oil was relatively ineffective against *T. mentagrophytes* at 1 × MIC (0.25 mg/mL), reducing the number of viable cells by 0.4-log_10_ after 8 h, but it eliminated the fungus (2.85-log_10_ reduction) after 1 h at both 4 and 16 × MIC.

### Electron microscopy

**Figure-3:**
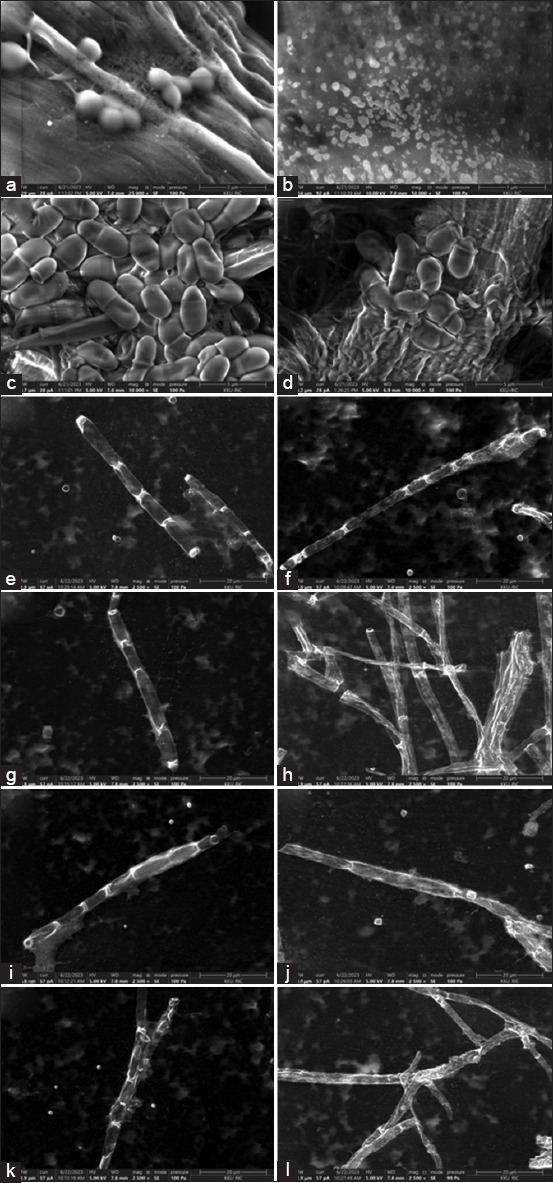
Morphological changes of microorganisms under environmental scanning electron microscopy after being treated with catnip essential oil: *Staphylococcus pseudintermedius* ATCC 49051 control (a) and treatment (b), *Malassezia pachydermatis* ATCC 14522 control (c) and treatment (d), *Microsporum canis* DMST 29297 control (e) and treatment (f), *Microsporum gypseum* DMST 21146 control (g) and treatment (h), *Trichophyton mentagrophytes* DMST 19735 control (i) and treatment (j), and *Microsporum gallinae* ATCC 90749 control (k), and treatment (l). Control=5% dimethyl sulfoxide solution. DMST=Department of Medical Sciences Thailand, ATCC=American Type Culture Collection.

Catnip essential oil inflicted obvious changes in the cellular morphology of all tested species, as detected by E-SEM (Figure-3). The *S. pseudintermedius* bacterium was particularly sensitive. After treatment with catnip essential oil, only irregularly shaped fragments (diameter, 0.2 μm) remained (Figure-3b) compared with the characteristic small clusters of spheroid cells (diameter, 0.5–1 m) were observed in the bacterial control group (Figure-3a). The untreated control *M. pachydermatis* cells appeared smooth and uniformly oval shaped (Figure-3c). Catnip essential oil causes the cells to shrink and the cell edges become wrinkled and rough, making them shriveled (Figure-3d). All untreated dermatophytes exhibited a characteristic filamentous appearance under E-SEM with rectangular well-defined cells within smooth filaments (Figures-3e, g, i, and k). Treatment with catnip essential oil resulted in filaments with a rough appearance, marked wrinkles, and disruption of the boundaries between individual cells within the filaments (Figures-3f, h, j and l).

### Cell membrane integrity

**Figure-4:**
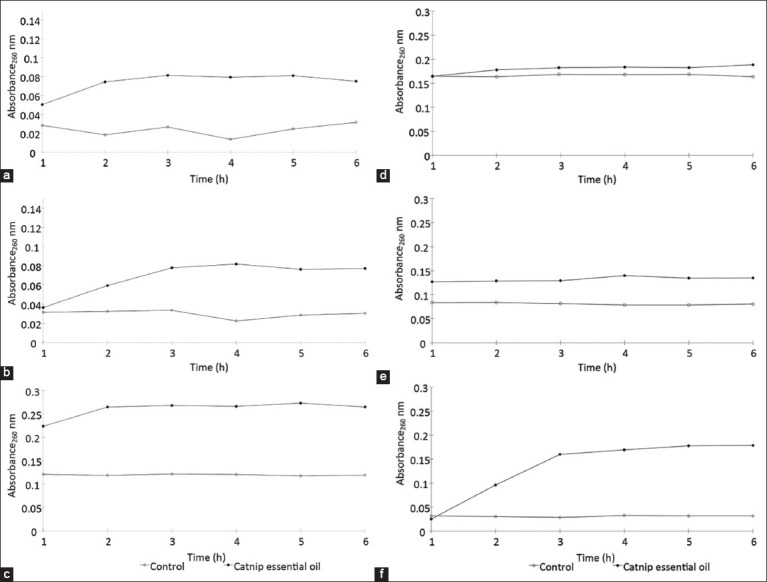
The effect of catnip essential oil on the integrity of cell membrane of catnip essential oil against *Staphylococcus pseudintermedius* ATCC 49051 (a), *Malassezia pachydermatis* ATCC 14522 (b), *Microsporum canis* DMST 29297 (c), *Microsporum gypseum* DMST 21146 (d), *Trichophyton mentagrophytes* DMST 19735 (e), and *Microsporum gallinae* ATCC 90749 (f). Control=5% dimethyl sulfoxide solution. DMST=Department of Medical Sciences Thailand, ATCC=American Type Culture Collection.

Cell membrane integrity was assessed by measuring the absorbance of intracellular nucleic acid components in extracellular supernatants at 260 nm (Abs_260_). The Abs_260_ values were lower for *S. pseudintermedius* (range 0.02–0.04 and 0.05–0.08, respectively) and *M. pachydermatis* (range 0.02–0.04 and 0.03–0.08, respectively) than for the dermatophytes *Mi. canis* (0.13 and 0.22–0.27), *Mi. gypseum* (0.16 and 0.14–0.19), *T. mentagrophytes* (0.09 and 0.13–0.14), and *Mi. gallinae* (0.03 and 0.03–0.18). In general, catnip essential oil-treated cells exhibited increased Abs_260_ values compared with control cells, indicating nucleic acid leakage from the cells. The catnip essential oil-induced increases in Abs_260_ values were time-dependent for *S. pseudintermedius* (Figure-4a), *M. pachydermatis* (Figure-4b), *Mi. canis* (Figure-4c), and *Mi. gallinae* (Figure-4f). In contrast, the maximum Abs_260_ value for *T. mentagrophytes* (Figure-4e) was reached at 1 h. In contrast, *Mi. gypseum* cells treated with catnip essential oil showed no difference in Abs_260_ values over time compared with control cells (Figure-4d). Thus, the results of the leakage study indicated that catnip essential oil compromised the integrity of cell membranes of all tested microorganisms except *Mi. gypseum*.

## Discussion

GC–MS analysis confirmed that our catnip essential oil contained *Z, E*- and *E, Z*-nepetalactone as the primary chemical constituents (totaling 96.78% of the peak area) along with *trans*-caryophyllene and caryophyllene oxide. Previously, Zomorodian *et al*. [13] showed that *E, Z*-nepetalactone comprised 55%–58% of the GC–MS peak area in their catnip essential oil prepared by hydrodistillation, followed by *Z, E*-nepetalactone (30.06%–31.2%), *trans*-caryophyllene (1.1%–2.7%), *β*-pinene (0.8%–1.64%), *α*-humulene (0.82%–0.92%), and other compounds. A recent study by Reichert *et al*. [9] reported slightly different chemical profiles of hydrodistilled catnip essential oils prepared from two distinct cultivars. According to the –GC–MS peak area, the CR3 cultivar contained 69.37% *E, Z*-nepetalactone, 27.45% *Z, E*-nepetalactone, 1.57% *trans*-caryophyllene, and 1.27% caryophyllene oxide. In contrast, the CR9 cultivar contained *Z, E*-nepetalactone as the highest constituent in the oil (83.55% of peak area), followed by caryophyllene oxide (5.79%), *trans*-caryophyllene (4.72%), *E, Z*-nepetalactone (1.68%), *β*-pinene (0.58%), and humulene (0.51%).

The highest MIC in the *in vitro* antimicrobial assay was 1 mg/mL, indicating that catnip essential oil has broad-spectrum antimicrobial activity against bacteria, yeast, and filamentous fungi that cause dermatitis in animal models. Time-kill tests showed that catnip essential oil had time- and concentration-dependent germicidal activity and that concentrations above 4 MIC could eradicate the tested microorganisms within 1 or 2 h. Previous studies have shown catnip to have some activity against pathogenic bacteria. Paşca *et al*. [11] found MIC values in the range of 0.78–12.5 mg/mL and agar diffusion inhibition zones of 8–11 mm for an ethanolic extract of catnip against bacteria that cause bovine mastitis, including *Staphylococcus aureus*, *Staphylococcus hyicus*, *Staphylococcus intermedius*, *Escherichia coli*, and *Bacillus subtilis*. Kim *et al*. [12] found that catnip essential oil (100 μL/disk) generated a 10 mm inhibition zone against *E. coli* in a disk diffusion assay. Catnip essential oil has also been shown to have strong antifungal activity against fungal pathogens of food and crops. At concentrations above 200 ppm, catnip essential oil prevented the growth of several genera of fungal crop pathogens [14]. It generated MIC values in the range of 0.25–1.0 mg/mL against several *Aspergillus* spp. food pathogens [13]. In the current study, the MIC and MFC values for catnip essential oil against *Mi. canis*, *Mi. gypseum*, and *T. mentagrophytes* were 4–32-fold lower than the respective values for the antifungal agent ketoconazole, indicating that catnip essential oil has potential for use as an alternative treatment against dermatophytes that cause canine skin infections.

E-SEM and leakage analysis performed in the current study identified the alteration of cell structures, especially cell membranes, as a potential mechanism for the antibacterial and antifungal activity of catnip essential oil. This activity is likely due to the nepetalactone component of catnip essential oil. Nepetalactones are iridoid monoterpenoids produced by *Nepeta* spp. that can disrupt membrane structures through their lipophilic properties, leading to membrane expansion and increased permeability, interference with membrane proteins, and alterations in ion transport mechanisms [22, 23].

## Conclusion

Catnip essential oil has potential as an alternative antimicrobial against a wide range of canine skin infection pathogens, including *S. pseudintermedius*, *M. pachydermatis*, *Mi. canis*, *Mi. gypseum*, *Mi. gallinae*, and *T. mentagrophytes*. However, this study has limitations as the antimicrobial activity testing does not encompass the microbial strains isolated from animal lesions and has not been studied in laboratory animals. There is a need for further studies to identify appropriate formulations, dosages, and clinical efficacy.

## Authors’ Contributions

GNB: Prepared the bacterial and fungal samples and contributed to the catnip essential oil extraction. RS and SS: Performed the catnip essential oil extraction and GC–MS analysis. PC: Prepared the plant samples and conducted the botanical identification. ET and JA: Performed the antimicrobial testing, SEM, and leakage study and drafted the manuscript. All authors have read, reviewed, and approved the final manuscript.
